# Inhibition of RNA binding to hepatitis C virus RNA-dependent RNA polymerase: a new mechanism for antiviral intervention

**DOI:** 10.1093/nar/gku632

**Published:** 2014-07-22

**Authors:** Abdelhakim Ahmed-Belkacem, Jean-François Guichou, Rozenn Brillet, Nazim Ahnou, Eva Hernandez, Coralie Pallier, Jean-Michel Pawlotsky

**Affiliations:** 1 Inserm U955, Hôpital Henri Mondor, 51 avenue du Maréchal de Lattre de Tassigny, 94010 Créteil, France; 2 Centre de Biochimie Structurale, Inserm U1054, CNRS UMR5048, Universités Montpellier 1 et 2, 29 rue de Navacelles, 34090 Montpellier, France; 3 Department of Virology, Hôpital Paul Brousse, 12 avenue Paul Vaillant Couturier, 94800 Villejuif, France; 4 National Reference Center for Viral Hepatitis B, C, and Delta, Department of Virology, Hôpital Henri Mondor, Université Paris-Est, 51 avenue du Maréchal de Lattre de Tassigny, 94010 Créteil, France

## Abstract

The hepatitis C virus (HCV) RNA-dependent RNA polymerase (RdRp) is a key target for antiviral intervention. The goal of this study was to identify the binding site and unravel the molecular mechanism by which natural flavonoids efficiently inhibit HCV RdRp. Screening identified the flavonol quercetagetin as the most potent inhibitor of HCV RdRp activity. Quercetagetin was found to inhibit RdRp through inhibition of RNA binding to the viral polymerase, a yet unknown antiviral mechanism. X-ray crystallographic structure analysis of the RdRp-quercetagetin complex identified quercetagetin's binding site at the entrance of the RNA template tunnel, confirming its original mode of action. This antiviral mechanism was associated with a high barrier to resistance in both site-directed mutagenesis and long-term selection experiments. In conclusion, we identified a new mechanism for non-nucleoside inhibition of HCV RdRp through inhibition of RNA binding to the enzyme, a mechanism associated with broad genotypic activity and a high barrier to resistance. Our results open the way to new antiviral approaches for HCV and other viruses that use an RdRp based on RNA binding inhibition, that could prove to be useful in human, animal or plant viral infections.

## INTRODUCTION

Hepatitis C virus (HCV) is a member of the *Hepacivirus* genus within the *Flaviviridae* family. HCV is a major causative agent of chronic liver disease, with over 170 million individuals chronically infected worldwide. Chronic HCV infection is responsible for chronic hepatitis which, in turn, leads to cirrhosis in ∼20% of cases and hepatocellular carcinoma at an incidence of 4–5% per year in cirrhotic patients (1). No prophylactic vaccine is available.

For the past 15 years, treatment of chronic hepatitis C has been based on the combination of pegylated interferon (IFN)-α and ribavirin (2). A number of new anti-HCV drugs, including protease inhibitors and various classes of inhibitors of HCV replication, have reached clinical development (3). IFN-free regimens yielding high HCV infection cure rates (over 90%) are likely to reach the market in 2014–2015 and onwards. These new treatment regimens will, however, be extremely costly and will generate multidrug resistance in patients who fail on therapy. They are unlikely to be available in the short- to mid-term in many areas of the world where therapeutic needs are high.

The RNA-dependent RNA polymerase (RdRp), or non-structural 5B (NS5B) protein, catalyzes HCV RNA replication, i.e. the synthesis of single-stranded positive-strand RNA genomes (4). As such, it is an obvious target for antiviral intervention. Two main groups of HCV RdRp inhibitors are at the pre-clinical to late clinical developmental stages, including nucleoside/nucleotide analogs (NI) and non-nucleoside inhibitors (NNI) (3). NNIs bind to one of the RdRp allosteric sites and this binding alters the 3D conformation of the enzyme, thereby impairing polymerase activity at the initiation step (5).

The 3D structure of HCV RdRp revealed a ‘right hand’ shape, including ‘fingers’, ‘palm’ and ‘thumb’ subdomains (6–8). Analysis of the crystal structure of the HCV RdRp, together with inhibition and binding studies with different classes of NNIs, identified 4 allosteric binding sites, including ‘thumb’ pocket I (thumb-1), ‘thumb’ pocket II (thumb-2), ‘palm’ pocket I (palm-1) and ‘palm’ pocket II (palm-2) (5). Thumb-1 is located at ∼30 Å of the active site, in the upper section of the thumb domain, adjacent to the allosteric guanosine triphosphate (GTP)-binding site (9). Thumb-1 ligands include benzimidazole and indole derivatives (10). Thumb-2 is a shallow hydrophobic pocket, located at the base of the thumb domain, next to thumb-1. Chemotypes of thumb-2 binders include thiophene (11), phenylalanine (12), dihydropyranone (13) and pyranoindole analogues (14). Palm-1 is situated in the inner thumb/palm domain, adjacent to the active site. Reported palm-1 ligands include benzothiadiazine, proline sulfonamide, benzylidene and acrylic acide derivatives (15,16). Finally, the palm-2 binding site resides in a large hydrophobic pocket within the palm domain that accommodates benzofuran inhibitors (17).

Silymarin is a mixture of flavonolignans (molecules with a flavonoid part and a lignan part) extracted from milk thistle (*Silybum marianum*). The principal components of silymarin are the diastereoisomers silibinin A and silibinin B in a roughly 1:1 ratio. A soluble formulation of silibinin hemisuccinate, Legalon SIL^®^ (Madaus-Rottapharm, Cologne, Germany), was shown to potently reduce HCV RNA levels in infected patients when administered intravenously (18,19). We previously demonstrated that silibinin's antiviral effect is at least partly explained by its ability to act as a direct non-nucleoside inhibitor of HCV RdRp activity (20). These findings have been confirmed, but other non-specific mechanisms may also play a role in the clinical effect of intravenous silibinin hemisuccinate (21–23).

The goal of this study was to identify natural flavonoids other than silibinin that efficiently inhibit HCV RdRp, identify their binding site and characterize their molecular mechanism of HCV RdRp inhibition.

## MATERIALS AND METHODS

### Chemical compounds

The flavonoids used in this work were purchased from Extrasynthèse (Genay, France). A non-nucleoside inhibitor of HCV-NS5BΔ21 belonging to the benzimidazole family, JT-16 [JT(2-[4-[[4-(acetylamino)-4′-chloro-[1,1’-biphenyl]-2-yl]methoxy]-phenyl]-1-cyclohexyl-1H-benzimidazole-5-carboxylic acid] was provided by the ViRgil-DrugPharm platform of the European Network of Excellence on Hepatitis and Influenza Virus Resistance ‘ViRgil’ (grant LSHM-CT-2004–503359 from the 6th Framework Program of the European Union).

### Expression and purification of recombinant HCV NS5BΔ21 RdRp

The HCV RdRps (NS5B protein) from reference strains H77 (genotype 1a), J4 (genotype 1b) and JFH1 (genotype 2a), truncated of their 21 C-terminal amino acids to ensure solubility (NS5BΔ21) and carrying a hexahistidine tag (His_6_-Tag) at their N-terminus, were expressed in *Escherichia coli* C41(DE3) and purified as previously described (20). Briefly, cultures were grown at 37°C for ∼1 h and induced with 1 mM isopropyl β-D-thiogalactoside for 4 h at 37°C. Cell pellets were re-suspended in a lysis buffer containing 50 mM NaH_2_PO_4_ (pH 8.0), 300 mM NaCl, 0.1% Triton X100, 0.525 mg/ml lysozyme, 0.1 U/μl desoxyribonuclease and Complete^TM^ Protease Inhibitor Cocktail Tablets (Roche Applied Science, Mannheim, Germany; one tablet for 10 purifications). After sonication, cell lysates were clarified by centrifugation, and chromatography was performed on a Ni-NTA column (Qiagen, Hilden, Germany). The bound protein was eluted in 1 ml fractions with a buffer containing 50 mM NaH_2_PO_4_ (pH 8.0), 500 mM NaCl and 250 mM imidazole. NS5BΔ21-enriched fractions were selected using a Bradford colorimetric assay, and HCV-NS5BΔ21 purity was determined by Coomassie-stained sodium dodecyl sulfate-polyacrylamide electrophoresis gel (SDS-PAGE) analysis. Purified NS5BΔ21 fractions were pooled and dialyzed against a buffer containing 5 mM Tris (pH 7.5), 0.2 M sodium acetate, 1 mM DTT, 1 mM ethylenediaminetetraacetic acid (EDTA) and 10% glycerol.

### HCV-NS5BΔ21 polymerase assay

The cell-free HCV-NS5BΔ21 polymerase assay is based on the real-time measurement of the amount of double-stranded RNA synthesized in the presence of HCV-NS5BΔ21, a homopolymeric RNA template (poly U or poly C, GE Healthcare, Chalfont St. Giles, UK) and the corresponding nucleotide, by means of an intercalating agent (SYBR^®^ Green, Applied Biosystems, Carlsbad, CA, USA), as previously described (20). For screening, HCV-NS5BΔ21 polymerase activity was quantified by the slope of fluorescence increase in the presence of 20 μM of the tested flavonoid compounds and compared with the control reaction containing no inhibitor. For each compound found to inhibit more than 50% of the HCV-NS5BΔ21 polymerase activity, the inhibitory concentration 50% (IC50), i.e. the compound concentration that inhibits polymerase activity by 50%, was calculated from the inhibition curve using a four-parameter logistic regression equation by means of Sigma Plot 10 software (Systat Software, San Jose, CA, USA). The reported values are the average of three independent measurements performed in duplicate. For kinetics analysis, initial reaction velocities were measured at different nucleotide (adenosine triphosphate (ATP) or GTP) and studied compound concentrations, without pre-incubation and in the presence of 50 μg/ml of the corresponding homopolymeric RNA template (Poly U and Poly C, respectively). The concentration of NS5BΔ21 (J4 strain, genotype 1b) in the reaction was 0.1 mg/ml. Kinetics data were graphically represented as double reciprocal plots. Data points were fitted by linear regression to identify the inhibition mode of the compound.

### RNA binding assay

RNA binding was studied by means of the electrophoretic mobility shift assay (EMSA) with minor modifications. Briefly, 0.5 μg/ml of HCV-NS5BΔ21 was incubated with 1 μg of a 500 bp RNA at 37°C for 30 min in 20 μl of a binding buffer containing 10 mM Tris pH 7.5, 1 mM EDTA, 100 mM KCl, 0.1 mM DTT, 5% vol/vol glycerol, 10 μg/ml bovine serum albumin (BSA), in the presence of increasing concentrations of the tested compound. Samples were then loaded onto a non-denaturing agarose gel. The gel was incubated 20 min in SYBR green EMSA Nucleic Acid Gel Stain (Invitrogen, Carlsbad, CA, USA). After washing, the gel was visualized under ultraviolet transillumination.

### HCV-NS5BΔ21-RNA enzyme-linked immunosorbent assay (ELISA) assay

Ninety-six-well plates were coated with streptavidin (100 μg/ml in 0.1 M NaHCO3, 50 μl/well) overnight at 4°C. The plates were washed six times with 200 μl phosphate-buffered saline (PBS)-T and blocked with 50 μl of 3% BSA and 0.1 μg/ml of streptavidin in PBS overnight at 4°C, then four times with 0.1% PBS-Tween. A mix of 5 pmol of biotinylated RNA and 0.5 μg of HCV-NS5BΔ21 previously incubated for 30 min at 37°C in a binding buffer containing 50 mM Tris (pH 7.5), 150 mM NaCl, 0.02 mg/ml yeast tRNA and 0.2 mg/ml BSA was added (50 μl/well) and incubated 1 h at room temperature. The plates were washed six times with 200 μl/well PBS-T, and the 6His mAB/HRP conjugate (Clontech, Mountain View, CA, USA) in PBS 1:1000 (50 μl/well) was added and incubated for 1 h at room temperature. The plates were washed six times with 200 μl/well PBS-T. A mix of enhanced chemiluminescence reagent was added (50 μl/well) and luminescence was measured.

### Assessment of antiviral activity in the replicon model

Huh7.5 cells harboring a Con1 genotype 1b subgenomic replicon were seeded at a density of 5000 cells per well in 96-well plates. The cells were treated with increasing concentrations of the tested compounds in Dulbecco's modified Eagle's medium (DMEM) containing 10% fetal bovine serum and 0.2% dimethylsulfoxid (DMSO) without G418 and cultured for 3 days. Total RNA was extracted using the RNeasy 96 kit (Qiagen, Valencia, CA, USA). HCV RNA levels were measured by means of a quantitative real-time polymerase chain reaction assay using the Taqman technology with HCV-specific primers (sense 5′- CGCCCAAACCAGAATACGA-3′ and antisense 5′- AGATAGTACACCCTTTTGCCAGATG-3′) and probe (5′-6-FAMCAATGTGTCAGTCGCG-TAMRA-3′) on an ABI 7003 device (Applied Biosystems, Foster City, CA, USA). Each data point represents the average of at least three replicates in cell culture. HCV RNA level reductions after treatment were assessed by comparing the level of HCV RNA in compound-treated cells to that of control cells treated with 1% DMSO. The effective concentration 50% (EC50), i.e. the compound concentration at which 50% of the maximal effect is achieved, was calculated using a four-parameter curve fitting method in the Sigma Plot 10 software (Systat Software, San Jose, CA, USA).

### Transient genotype 1b subgenomic replicon assay

The HCV-N genotype 1b subgenomic replicon containing a luciferase reporter gene (24) was transfected into Huh7.5 cells by means of Mirus tranfecting agent (Mirus Bio, Madison, WI, USA) in 96-well plates, according to the manufacturer's instructions. Four hours post-transfection, control wells were harvested for assessment of transfection efficiency by a luciferase assay. Transfected cells were grown in DMEM (Invitrogen) supplemented with 10% fetal bovine serum, 50 IU/mL penicillin, 100 μg/mL streptomycin, 0.1 μg/mL fungizone, in the absence or presence of increasing concentrations of the tested compound (1% DMSO final concentration) for 72 h. The medium was then removed and the plates were washed with 100 μl of PBS per well. A total of 30 μl of Lysis buffer (Promega, Madison, WI, USA) was added into each well and the plates were incubated for 5 min at room temperature. Luciferin solution was added, and luciferase activity was measured with a Berthold luminometer (Pforzheim, Germany) and normalized to the total protein concentration.

### Assessment of HCV resistance

Huh7.5 cells containing the genotype 1b bicistronic replicon I389-neo/NS3–3’/5.1 (25) were sequentially treated with 20, 50 and 75 μM of the compound in the presence or in the absence of 2 mg/ml of G418 for 11, 21 and 31 days, respectively. HCV-RNA was then extracted at day 11, 32 and 63 and the full-length NS5B region was sequenced.

### Western blot analysis

HCV Con1 replicon-harboring cells were seeded at a density of 90 000 cells per well in 6-well plates. The cells were treated with increasing concentrations of the tested compound (0–100 μM) in DMEM containing 10% fetal bovine serum and 1% DMSO without G418 and cultured for 3 days. Cell lysates were prepared, the proteins were quantified (BCA Protein Assay, Thermo Scientific, Rockford, IL, USA) and 10 μg of protein was separated on a 4–12% SDS-PAGE. NS5B and actin were detected using specific monoclonal antibodies (Enzo Life Sciences, Firmingdale, NY, USA and Sigma Aldrich, Saint Louis, MO, USA, respectively).

### J6/JFH1 infection in cell culture

The plasmid pFL-R-luc2A-ubi-J6/JFH1, containing the full-length cDNA of the chimeric J6/JFH1 HCV genotype 2a clone and the Renilla luciferase gene, was used to generate infectious HCV particles (HCVcc) in Huh7.5 cell culture. Huh7.5 cells were seeded in 48-well plates at a density of 10 000 cells/well, infected 24 h later with 100 μl of HCVcc and incubated overnight at 37°C. After incubation, the supernatants were removed and J6/JFH1-infected cells were washed with fresh medium. Increasing concentrations of the tested compounds were added in a medium containing 1% DMSO, and the cells were incubated 72 h at 37°C. Then, the cells were washed once with Dulbecco's PBS (Invitrogen), and 75 μl Renilla lysis buffer (Promega) was added to each well. Note that 20 μl of lysate was mixed with the luciferase assay substrate, as specified by the manufacturer. Luciferase activity was measured for 10 s in a Berthold luminometer.

### Cytotoxicity assay

Exponentially growing Huh7 and HEK293 cells were trypsinized and plated (2000 and 1000 cells/well, respectively) in 96-well microplates in DMEM glutamax-II supplemented with 10% fetal bovine serum and allowed to attach for 24 h at 37°C in the presence of 5% CO_2_. A total of 200 μl of increasing concentrations of the compound diluted in DMEM with 0.5% DMSO was added and the cells were incubated for 3 days at 37°C in the presence of 5% CO_2_. Cell viability was assessed using a 3-(4,5-dimethylthiazol-2-yl)-2,5-diphenyltetrazolium bromide colorimetric assay. The 50% cytostatic concentration (CC50), defined as the compound concentration that inhibited proliferation of exponentially growing cells by 50%, was calculated using a four-parameter logistic equation using Sigma Plot 10 software. Each value is the result of three experiments performed in quadruplicate.

### Determination of the structure of the HCV-NS5BΔ21 complex with the inhibitory compound

The HCV RdRp from reference strain J4 (genotype 1b), truncated of its 21 C-terminal amino acids to ensure solubility (NS5BΔ21) and carrying an hexahistidine tag at its N-terminus, was purified by Ni-NTA affinity chromatography, as described above. The combined peak fractions were loaded onto an S75 column equilibrated with 50 mM Tris (pH 8.0), 400 mM NaCl, 2 mM dithiothreitol and 5% glycerol. NS5BΔ21-enriched fractions were selected using a Bradford colorimetric assay, and HCV-NS5BΔ21 purity was determined by Coomassie-stained SDS-PAGE analysis. The combined peak fractions were concentrated to 20 mg/ml.

Apo NS5BΔ21 crystals were grown using the hanging drop vapor diffusion method at 18°C. Drops were formed by mixing 1 μl of protein with an equal volume of mother liquor containing 0.2 M sodium acetate (pH 5.1), 16% (w/v) PEG 4000, 2 mM DTT and 5% glycerol and equilibrated over 500 μl of the same solution. Crystals generally formed within a few days. Soaking of the NS5BΔ21 crystals was performed for 2 h using 0.2 μl of a solution containing 100 mM of the compound in DMSO. Prior to data collection, the crystals were cryoprotected by a brief immersion in oil. X-ray diffraction data were collected at the European Synchrotron Radiation Facility, Grenoble, France, on the beamline ID14–4 (λ = 0.93340 Å). Data were integrated and processed using MOSFLM and SCALA from the CCP4 suite (26). The crystals belong to the space group P21212, with two monomers in the asymmetric unit. The structures were solved by molecular replacement using Protein Data Bank (PDB) entry 2HAI as the search model. Bound ligand was manually identified and fitted into Fo–Fc electron density using Coot. The structure was refined by rounds of rebuilding in Coot and further refined using Refmac from the CCP4 suite. Figures were generated with PyMOL (DeLano Scientific). Diffraction and refinement statistics are summarized in Supplementary Table S1.

## RESULTS

### Flavonoids inhibit HCV RdRp activity in an enzyme assay and quercetagetin is the most potent inhibitor

**Figure 1.**
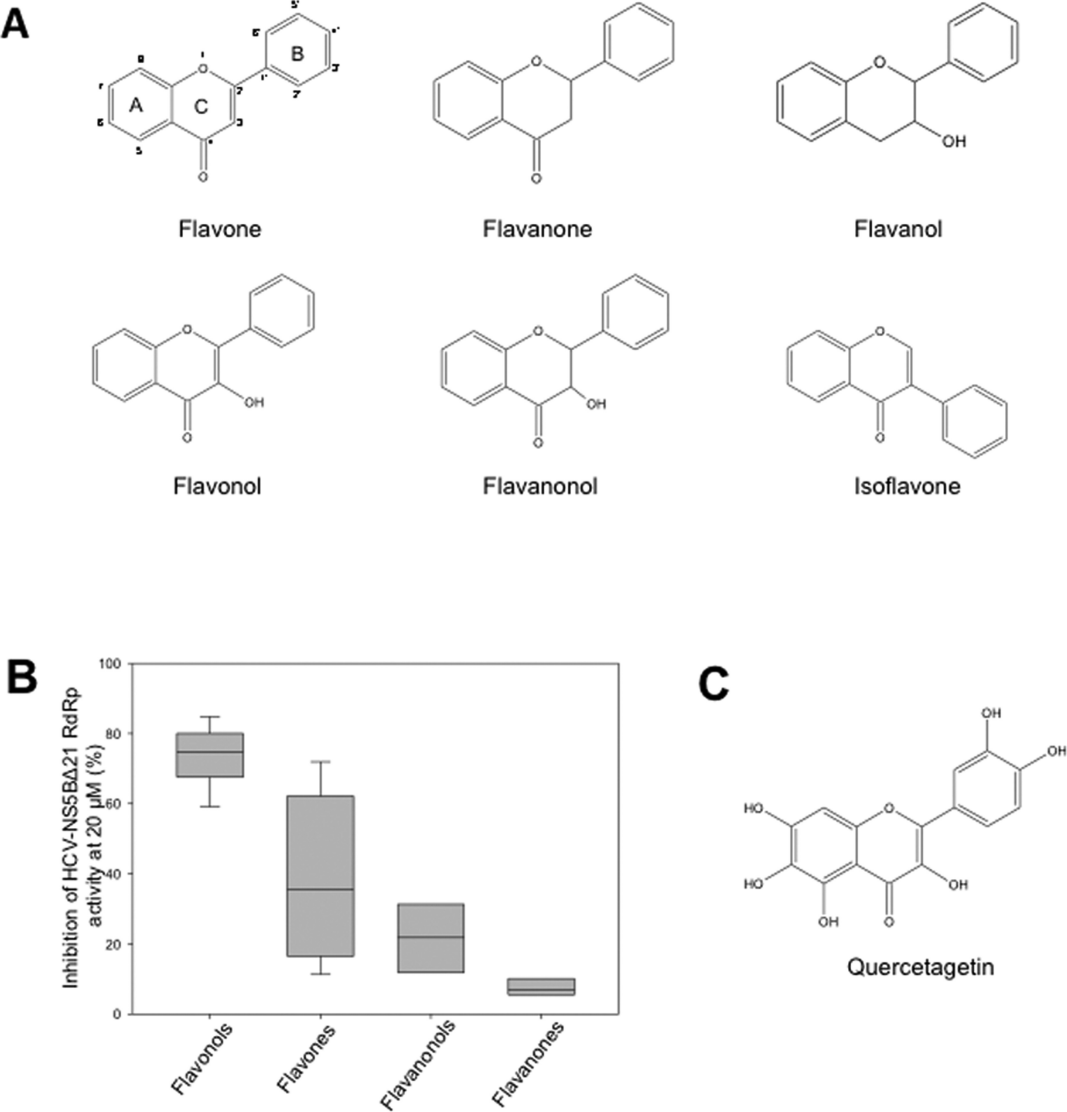
(A) Chemical structure of representative compounds from the six flavonoid subfamilies tested. (B) Box-plot distribution of HCV-NS5BΔ21 RdRp activity inhibition by members of the flavonol, flavone, flavanonol and flavanone subfamilies at 20 μM. (C) Chemical structure of quercetagetin.

Forty-four compounds from different subfamilies of the flavonoid family, including flavonol, flavone, flavonoid glycosides, flavanon(ol), isoflavones and flavanol (Figure 1A), were tested for their ability to inhibit the polymerase activity of HCV-NS5BΔ21 from the genotype 1b reference strain J4 in a cell-free enzyme assay. The most potent inhibitors were from the flavonol family, but antiviral activities overlapped between compounds from the different families (Figure 1B and Table 1). The most potent inhibitor was quercetagetin (Figure 1C), a member of the flavonol subfamily, which inhibited HCV-NS5BΔ21 polymerase activity by 85.6% at a 20 μM concentration. Quercetagetin was thus chosen as our hit compound for subsequent experiments.

**Table 1.** Slope of fluorescence increase and percent inhibition of HCV RdRp (HCV-NS5BΔ21) by 44 members of the flavonoid family at the concentration of 20 μM in an enzyme assay

Class	Compound name or chemical name	Slope of fluorescence increase	% HCV-NS5BΔ21 inhibition
No treatment (control)		62.0	0
			
Flavonols	Quercetagetin	8.9	85.6
	Quercetin	10.1	83.7
	Galangin	11.9	80.8
	Myricetin	12.9	79.2
	Isorhamnetin	13.9	77.6
	Kaempferol	15.7	74.7
	Gossypetin	15.8	74.6
	7-hydroxyflavonol	17.8	71.3
	Datiscetin	18.3	70.5
	Morin	19.0	69.3
	Fisetin	21.2	65.8
	5-Deoxykampferol	22.4	63.9
	Robinetin	27.4	55.9
			
Flavones	Luteolin	11.4	81.7
	Tricetin	18.9	69.5
	Scutellarein	20.2	67.4
	Apigenin	23.3	62.5
	Baicalein	23.7	61.8
	5.7.8-Trihydroxyflavone	32.6	47.4
	3’.4’-Dihydroxyflavone	33.9	45.4
	6.7 Dihydroxyflavone	38.8	37.4
	3’.4’.7-Trihydroxyflavone	40.1	35.4
	3’.4’.7.8-Tetrahydroxyflavone	42.9	30.9
	7.8 Dihydroxyflavone	42.9	30.8
	7.4’-Dihydroxyflavone	46.1	25.7
	6-Hydroxyflavone	50.6	18.5
	Quercetin 3.7.3’.4’ tétramethylether	53.0	14.5
	5-hydroxyflavone	53.0	14.5
	Flavone	53.9	13.1
	Quercetin 3.5.7.3’.4’ pentamethylether	59.6	4.0
			
Flavonoid glycosides	Quercetagetin-7-O glucoside	35.7	42.5
	Rutin	43.2	30.3
	Hyperoside	44.6	28.1
	Quercetin-3-O glucopyranoside	46.0	25.9
			
Flavanon(ol)s	Fustin	53.0	14.5
	7-Hydroxyflavanone	54.3	12.5
	Dihydrorobinetin	55.2	11.0
	4’-Hydroxyflavanone	56.3	9.3
	Flavanone	57.4	7.5
	Liquiritigenin	58.3	6.1
	Naringenin	58.3	6.0
	6-Hydroxyflavanone	59.5	4.1
			
Isoflavones	Genistein	53.9	13.1
			
Flavanols	DL-Catechin	51.1	17.7

### Quercetagetin inhibits HCV genotypes 1a, 1b and 2a replication in an enzyme assay

**Figure 2.**
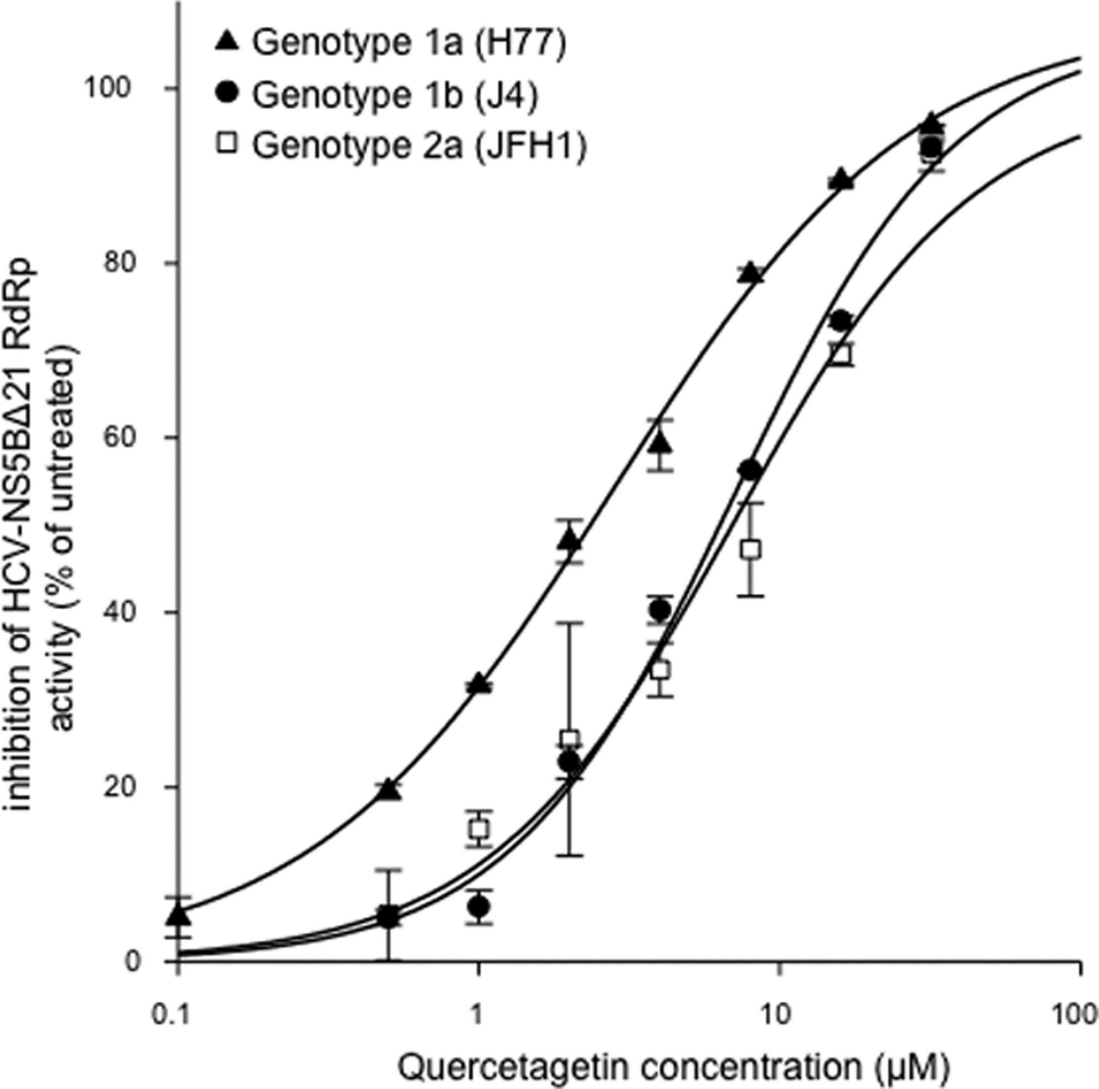
Concentration-dependent inhibition of HCV-NS5BΔ21 RdRp activity from genotype 1a (H77 strain), genotype 1b (J4 strain) and genotype 2a (JFH1 strain) in an enzyme assay. The mean ± SD of three independent experiments performed in duplicate are shown.

Figure 2 shows the inhibition of HCV-NS5BΔ21 polymerase activity as a function of quercetagetin concentration in a cell-free enzyme assay using the enzyme from 3 reference HCV strains of different genotype/subtypes. Quercetagetin's IC50s were 2.8 ± 0.4 μM for genotype 1a (strain H77), 4.3 ± 0.7 μM for genotype 1b (strain J4) and 6.1 ± 1.6 μM for genotype 2a (strain JFH1).

### Quercetagetin inhibits HCV genotypes 1b and 2a replication in cell culture

**Figure 3.**
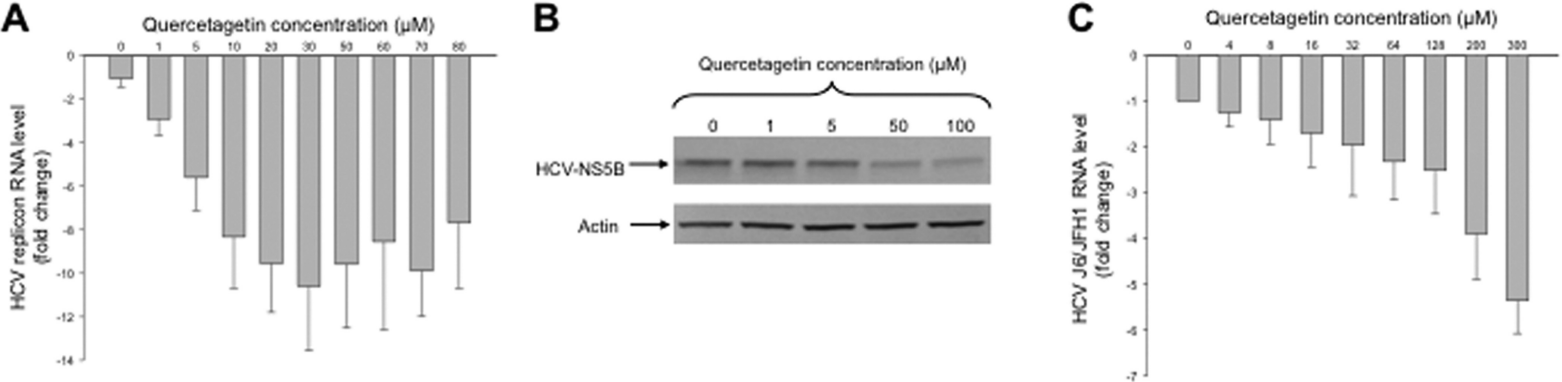
(A) Concentration-dependent inhibition of HCV genotype 1b subgenomic replicon replication by quercetagetin in Huh7.5 cells. (B) Western-blot analysis showing the concentration-dependent inhibition of HCV NS5B protein expression by quercetagetin in Huh7.5 cells stably expressing a Con1 genotype 1b subgenomic replicon. Actin was used as a control. (C) Concentration-dependent inhibition of the chimeric genotype 2a/2a infectious J6/JFH1 strain (HCVcc) replication by quercetagetin in Huh7.5 cells. Mean ± SD values of three experiments performed in triplicate are shown.

As shown in Figure 3A, quercetagetin inhibited replication of the HCV genotype 1b subgenomic replicon Con1 in a dose-dependent manner. Its EC50 was 5.4 ± 3.3 μM. In addition, western blot analysis carried out in Huh7.5 cells stably expressing an HCV genotype 1b replicon treated for 72 h with increasing concentrations of quercetagetin showed that quercetagetin inhibited NS5B protein expression in a dose-dependent manner (Figure 3B).

Figure 3C shows that quercetagetin also inhibited replication of the genotype 2a infectious J6/JFH1 strain in a dose-dependent manner in Huh7.5 cells, with an EC50 of 40.2 ± 17.7 μM in this model, eight times higher than its EC50 in the genotype 1b subgenomic replicon model.

### Quercetagetin inhibits HCV replication by inhibiting RNA binding to the RdRp

To investigate the mode of inhibition of quercetagetin, its capacity to inhibit HCV RdRp activity was assessed in the genotype 1b enzyme assay in the presence of increasing concentrations of ATP or GTP and the corresponding RNA homopolymeric matrix (poly-U or poly-C, respectively). Double-reciprocal plots of initial velocities demonstrated non-competitive inhibition (Supplementary Figure S1A and B). In addition, no abortive or prematurely terminated products were generated in elongation reactions in the presence of quercetagetin (data not shown).

**Figure 4.**
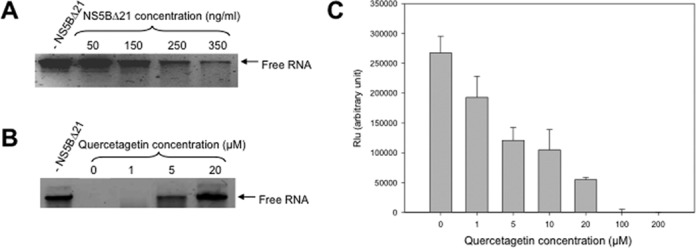
(A) RNA binding assay with increasing concentrations of HCV-NS5BΔ21 incubated 30 min with 1 μg of RNA, then loaded onto a non-denaturing agarose gel. (B) RNA binding assay with 500 ng/ml of HCV-NS5BΔ21 incubated 30 min with 1 μg of RNA in the presence of increasing concentrations of quercetagetin, then loaded onto a non-denaturing agarose gel. The control contained no HCV-NS5BΔ21. (C) Dose-dependent inhibition of RNA binding to HCV-RdRp by quercetagetin in a quantitative HCV-NS5BΔ21-RNA ELISA assay.

We then measured the amount of free RNA in the presence of increasing concentrations of purified HCV-NS5BΔ21. As expected, the amount of free RNA decreased in an HCV-NS5BΔ21 concentration-dependent manner, as a result of RNA binding to the enzyme (Figure 4A). At a fixed concentration of HCV-NS5BΔ21 that binds most of the free RNA, the amount of free RNA increased in a quercetagetin concentration-dependent manner (Figure 4B). In addition, we quantified quercetagetin inhibition of RNA binding by means of an HCV-NS5BΔ21-RNA ELISA assay. This experiment confirmed that quercetagetin inhibits RNA binding to the HCV RdRp in a dose-dependent manner, with an EC50 of the same order as that in the enzyme assay (Figure 4C).

Finally, an RNA duplex fluorescence assay was used to assess whether quercetagetin could directly interact with RNA. As shown in Supplementary Figure S2, RNA duplex formation was not inhibited in the presence of 20 μM of quercetagetin, ruling out the possibility that quercetagetin binds to RNA.

Altogether, these results indicate that quercetagetin inhibits RNA binding to the HCV RdRp through a direct interaction with the enzyme.

**The X-ray crystallographic structure of the NS5BΔ21/quercetagetin complex confirms that quercetagetin inhibits RNA binding**

**Figure 5.**
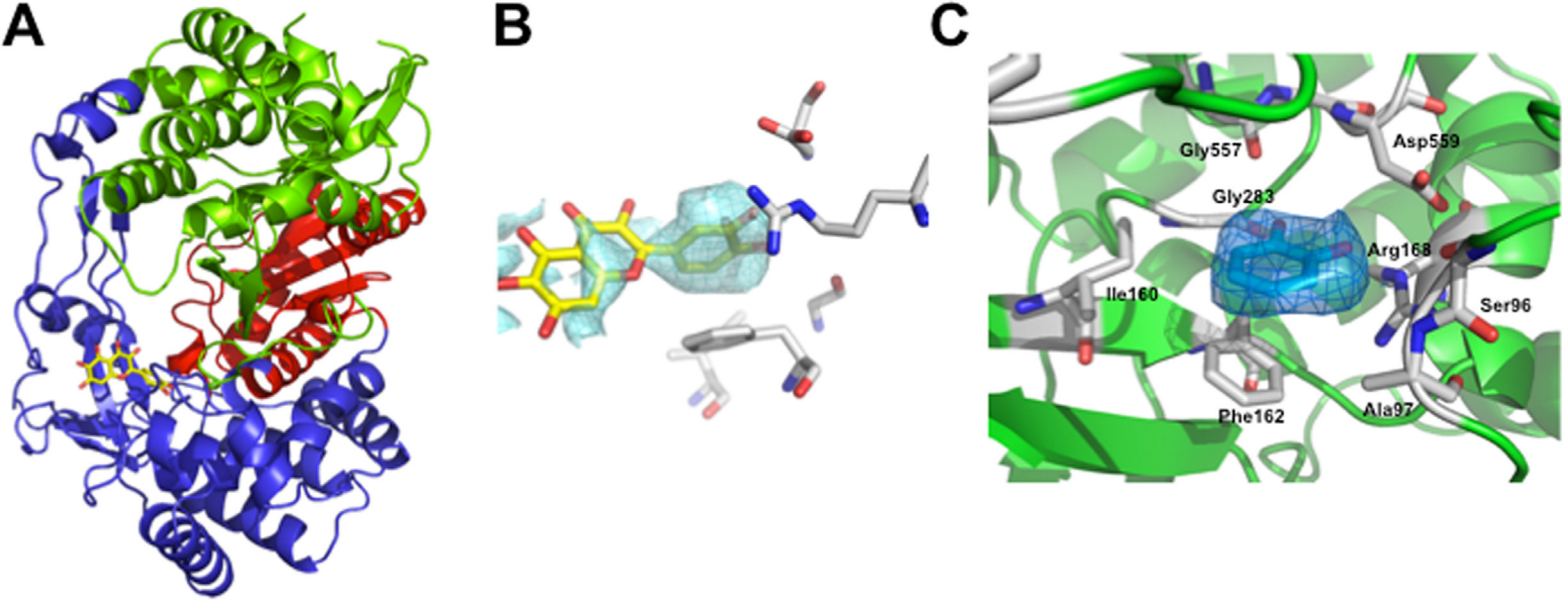
X-ray structure of the HCV-NS5BΔ21-quercetagetin complex at a resolution of 2.57 Å in ribbon representation (palm in red, thumb in green and fingers in blue) with quercetagetin in stick representation. (B) 2mFo-DFc map contoured at a 1 σ level of quercetagetin before refinement. (C) 2mFo-DFc map contoured at a 1 σ level of the B ring of quercetagetin after refinement. The figures were generated by means of Pymol software.

The X-ray crystal structure of quercetagetin bound to the HCV RdRp was solved by means of soaking experiments in apo crystals of NS5BΔ21. A crystallographic structure was obtained at a resolution of 2.57 Å (PDB 4OOW; Supplementary Table S1). The structure displayed the typical right-hand shape of an RNA polymerase (Figure 5A), with the palm, thumb and fingers subdomains organized around a central cleft defining the active site.

Quercetagetin was found to bind to the fingers and C-terminal domains of HCV RdRp, at the entrance of the RNA template tunnel. The B ring of quercetagetin was clearly apparent in the electron density maps, whereas the orientation of the A and C rings was proposed based on partial density only, because this part of the molecule adopted several conformations (Figure 5B). The occupancy for quercetagetin in the final structure was 100% and no other regions of positive density were identified by the electronic density maps. Quercetagetin was fitted into the same binding pocket by both manual placement and the ARP/Warp macromolecular model building software. In addition, among 10 poses generated by molecular docking analysis with Plant software, one perfectly fitted quercetagetin in the same binding pocket (data not shown).

Superimposition of common backbone atoms with the previously reported apopolymerase structure of NS5B (PDB 1QUV (7)) gave root mean square deviation values of 0.59 Å, suggesting that quercetagetin binding does not involve significant conformational adjustments of the protein. The structure showed that quercetagetin binds closely to residues D559, G557, G283, R168, F162, I160, A97 and S96, with its B ring within a 2.9–4.0 Å distance of these amino acids (Figure 5C), making a π-stacking interaction with the side chain of F162 and one hydrogen bond with G283.

**Figure 6.**
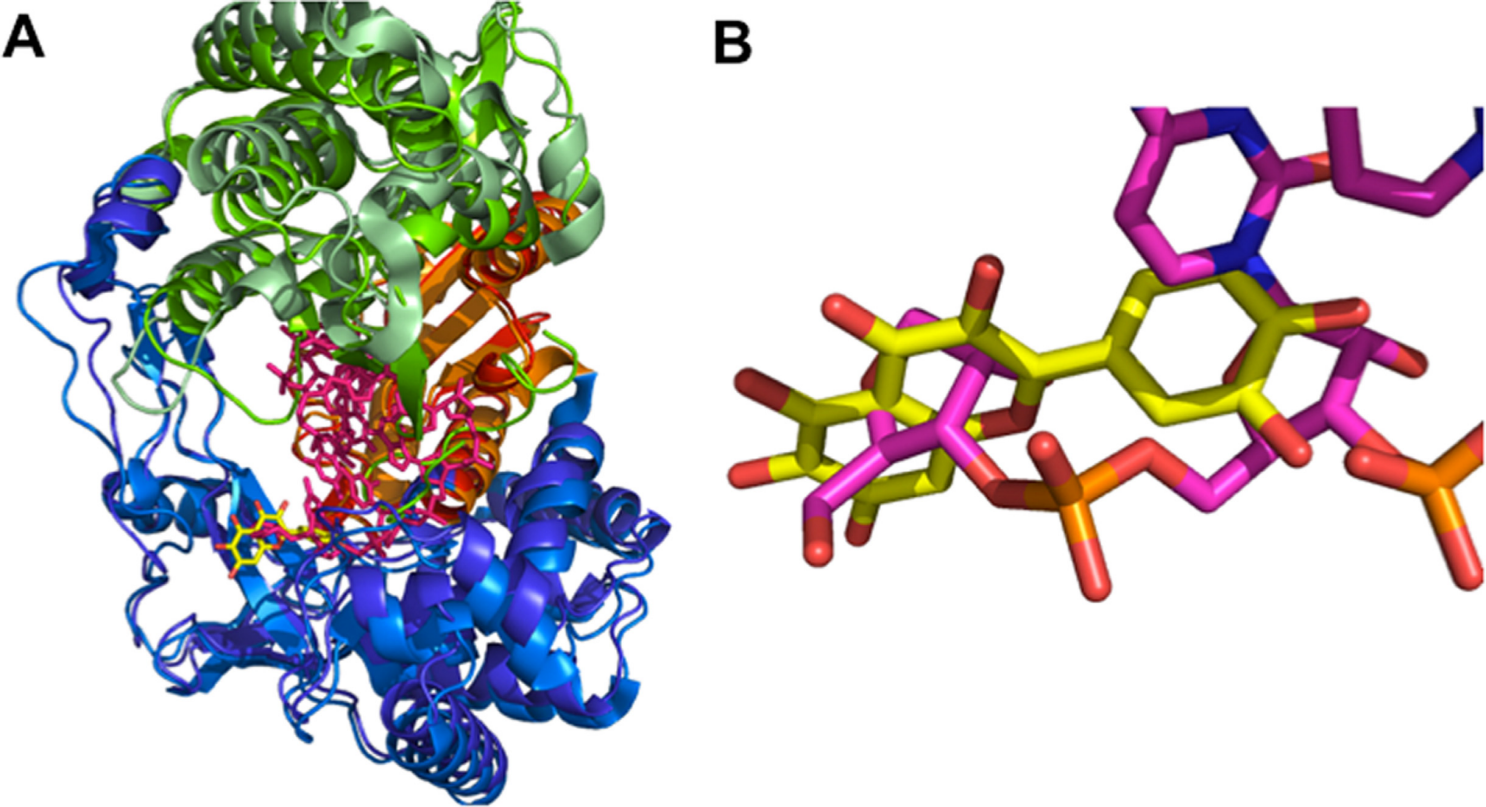
Superimposition of the HCV-NS5BΔ21-quercetagetin complex with the HCV-NS5BΔ21-RNA complex (PDB code 4E7A) in ribbon representation (palm in red and orange, thumb in green and pale green, fingers in blue and marine). Quercetagetin and RNA are in stick representation (yellow and pink, respectively). (A) Superimposition of the global structures. (B) Zoom on the quercetagetin binding domain.

Finally, comparison with the recently published structure of HCV RdRp in a complex with primer-template RNA (PDB 4E7A and 4E78 (27)) suggests that quercetagetin interferes with RNA binding (Figure 6A and B), a result in keeping with our RNA binding experiments. Indeed, the residues involved in the binding of the RNA template's pairing nucleotide ribose were also involved in binding the B ring of quercetagetin. That was the case of the strictly conserved G283 residue which interacts with the 2′-hydroxyl of the template RNA ±1 ribose and makes a hydrogen bond with the B ring of quercetagetin, and of the highly conserved F162 residue which stacks both the sugar of the RNA template pairing nucleotide and the B ring of quercetagetin at its top.

### The mechanism of antiviral action of quercetagetin is associated with a high barrier to resistance

**Figure 7.**
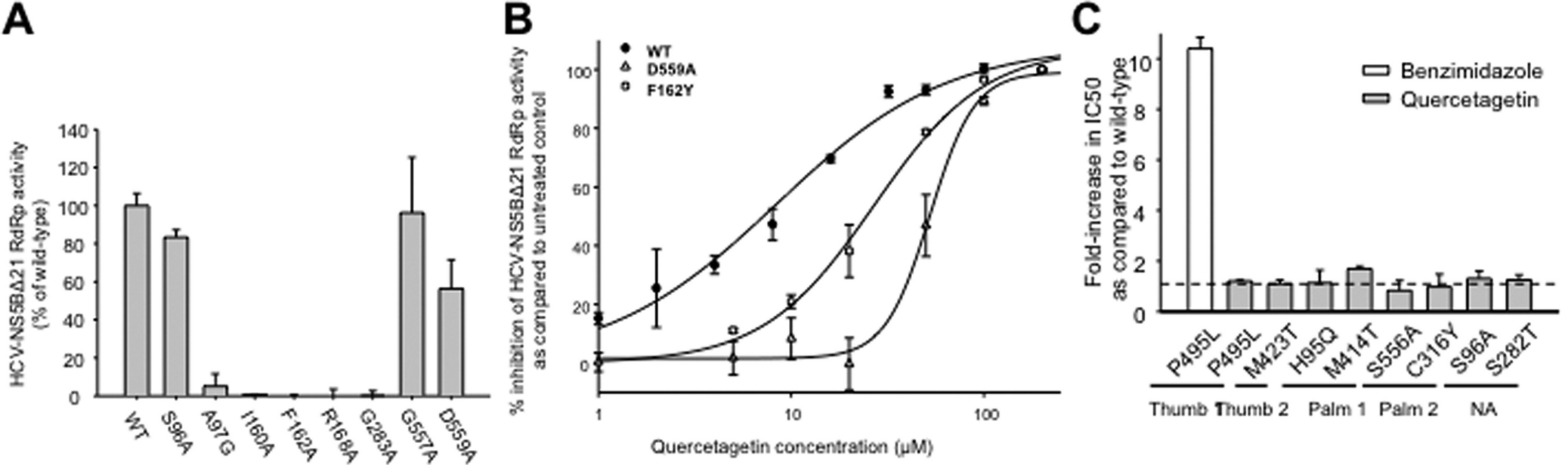
(A) RdRp activity of HCV-NS5BΔ21 mutants relative to wild type in a cell-free enzyme assay. (B) Susceptibilities of the D559A and F162Y HCV-NS5BΔ21 RdRp mutants to quercetagetin relative to wild type in a cell-free enzyme assay. (C) Fold-increase in IC50s relative to wild type of mutants harboring the P495L, M423T, H95Q, M414T, S556A, C316Y, S96A and S282T substitutions, known to confer resistance to other NNI classes. Benzimidazole, a thumb-1 inhibitor, was used as a control. The mean ± SD of three independent experiments performed in duplicate is shown.

We aimed to assess whether substitutions at sites predicted to interact with quercetagetin are compatible with viable catalytic activity and, if so, whether these substitutions confer resistance to quercetagetin. For this, the S96, A97, I160, F162, R168, G283, G557 and D559 residues, which are close to the B ring of quercetagetin, were replaced by an alanine or a glycine residue. As shown in figure 7A, NS5B variants bearing A97G, I160A, F162A, R168A or G283A substitutions failed to retain significant catalytic activity. Variants harboring the S96A and D559A substitutions had RdRp activities reduced by 17% and 44%, respectively, relative to wild type, whereas the G557A variant displayed RdRp activity comparable to the wild type. Susceptibility of the three catalytically active mutants S96A, G557A and D559A to quercetagetin was assessed. No change was observed for S96A and G557A, whereas susceptibility of the D559 variant was reduced 7-fold in the enzyme assay (Figure 7B). In addition, F162Y, a more conservative substitution that makes a π-stacking interaction with the B-ring of quercetagetin, was engineered. The corresponding variant exhibited 33% of wild-type catalytic activity at baseline, which was reduced by 3-fold by quercetagetin, a result in keeping with the proposed mechanism of action of this compound (Figure 7B).

The same amino acid substitutions were introduced in a transient genotype 1b HCV-N subgenomic replicon by means of site-directed mutagenesis. None of these replicons were viable (replication capacity <2% of wild type), except that harboring an S96A substitution that replicated at only ∼20% of wild type (data not shown). Replication was too low to assess quercetagetin resistance with this replicon variant.

Resistance selection experiments were performed in replicon-containing Huh7 cells treated with increasing concentrations of quercetagetin for 63 days. The only amino acid change observed was a V116I substitution at day 63. This substitution did not reduce catalytic efficiency nor quercetagetin susceptibility in the enzyme assay and in a genotype 1b subgenomic replicon into which it had been introduced by means of site-directed mutagenesis (data not shown).

Finally, amino acid changes known to confer resistance to the known groups of NI and NNI inhibitors of HCV RdRp were introduced in the HCV-NS5BΔ21 enzyme assay. They included P495L (thumb-1 inhibitors), M423T (thumb-2 inhibitors), H95Q and M414T (palm-1 inhibitors), S556A and C316Y (palm-2 inhibitors) and S96A and S282T (nucleoside/nucleotide analogues). None of these substitutions altered HCV RdRp susceptibility to quercetagetin (Figure 7C).

Altogether, these results show that the unique mode of antiviral action of quercetagetin is associated with a high barrier to resistance.

### Quercetagetin is not cytotoxic at effective concentrations

Quercetagetin cytotoxicity was assessed in two different human cell lines, including Huh7 and HEK293 cells. The selective index (CC50/IC50) was 32.9, indicating that quercetagetin is not cytotoxic at its inhibitory concentration (data not shown).

## DISCUSSION

Our *in vitro* enzyme assay based on RNA duplex formation in the presence of HCV-NS5BΔ21 allowed us to identify the flavonols, and among them quercetagetin, as the most potent inhibitors of HCV RdRp among the flavonoids tested. Quercetagetin's IC50 was of the order of 2.8–6.1 μM in this assay, nearly identical for genotypes 1b, 1a and 2a. It was of the same order in a genotype 1b subgenomic replicon in cell culture, but ∼10 times higher in the infectious genotype 2a model. This difference could be due to the presence of structural proteins in the latter model, that may interfere with quercetagetin RdRp inhibition. Our observation that quercetagetin was 19 times more active than silibinin against HCV genotype 1b RdRp and comparison of the chemical structures of the two compounds (data not shown) suggests that the lignan part of silibinin impairs its anti-RdRp activity.

We aimed at understanding the molecular mechanisms involved in quercetagetin inhibition of HCV RdRp. Our results suggested that quercetagetin acts as a non-nucleoside inhibitor of HCV RdRp. Four groups of NNIs have been described thus far, including thumb-1 and -2 and palm-1 and -2 inhibitors (5). They bind to their respective allosteric sites and alter the enzyme 3D structure, making it non-functional. Compounds belonging to these classes select distinct, although sometimes overlapping resistance-associated substitutions (3,28). Thus far, no crystallographic complexes have been generated that indicate that the ‘fingers’ subdomain of the HCV RdRp can act as a binding site for allosteric inhibitors.

We describe here, for the first time, a non-nucleoside HCV RdRp inhibitor that binds to the fingers domain, at the entrance of the RNA template tunnel. Our crystallographic structure of the NS5B protein in complex with quercetagetin indeed revealed that the B ring of this compound makes a hydrogen bond with the strictly conserved G283, and a π-stacking interaction with F162, which is conserved as a Tyr or Phe. Interestingly, G283 and F162 have been recently reported to be involved in an interaction with the 2′-hydroxyl and the sugar nucleobase of the template strand pairing nucleotide, respectively (27). Moreover, quercetagetin was located at less than 4 Å of the highly conserved S96, A97, I160 and R168 residues, which have been shown to be involved in the recognition and positioning of the RNA template strand (27). Superimposition of our crystallographic structure with the recently published crystallographic structure of HCV NS5B in complex with primer-template RNA (27) suggested that quercetagetin interferes with the ability of the enzyme to interact with RNA. To confirm this hypothesis, we titrated free RNA in the presence of HCV RdRp and increasing concentrations of quercetagetin and showed that quercetagetin indeed impairs RNA binding to the polymerase in a dose-dependent manner.

A number of new HCV drugs have reached clinical development and several therapeutic options based on all-oral combinations of antivirals will be available in the short- to mid-term future, in which NNIs play an important role (3). Recent results have shown that high rates of HCV clearance can be achieved with these regimens. However, still 5–10% of patients experience treatment failure and this number is likely to be substantially higher in the clinical setting, especially in the most difficult-to-treat patient populations. Patients who fail on these treatment regimens have been shown to harbor multidrug resistant viruses, and reversion to wild type may take years after treatment withdrawal. Thus, new treatment options are needed. An active search for second- or third-generation inhibitors with pangenotypic activity and a very high barrier to resistance used as backbones of future combination strategies is ongoing.

Most NNIs developed thus far are highly specific for genotype 1 and they have a low barrier to resistance (5). This is due to the fact that conservatory constraints on the corresponding regions are lower than on critical functional regions, such as the catalytic site. Thus, sequences differ between genotypes and natural polymorphisms at key binding positions are frequent. Due to the conservation of the residues at the quercetagetin binding site across HCV genotypes, this new NNI binding site has the potential for pangenotypic activity. Although genotypes 3–6 were not tested in this study, our results showing that quercetagetin equally inhibited genotypes 1a, 1b and 2a in the enzyme assay support this hypothesis.

Not surprisingly, substitution of highly conserved residues S96, A97, I160, F162, R168 and G283 that interact with quercetagetin abolished the replication of subgenomic replicons in cell culture. This suggests that compounds that target this region have the potential for a high barrier to resistance. Resistance selection experiments in replicon-containing Huh7 cells confirmed this hypothesis. The only amino acid change observed during these experiments was a V116I substitution that did not reduce quercetagetin susceptibility *in vitro*. Only substitution of the D559 residue was found to decrease HCV RdRp susceptibility to quercetagetin in our enzyme assay. The D559G substitution has been reported to confer *in vitro* resistance to A-837093, a non-nucleoside palm-1 inhibitor (29). In our experiments, substitutions at position 559 were associated with a dramatic impairment of replication capacities in the enzyme assay and in the replicon system. Whether mutations at these positions can be selected and be fit *in vivo* upon administration of compounds that share quercetagetin's mode of action remains to be determined. Finally, no cross-resistance with the four classical families of allosteric NNIs was found in our experiments. Altogether, these findings confirm the high barrier to resistance of quercetagetin and compounds sharing its binding site and mode of action, at least *in vitro*.

In conclusion, we identified quercetagetin, a natural compound belonging to the flavonol subfamily of flavonoids, as a potent inhibitor of HCV replication *in vitro.* By means of virological and structural approaches, we showed that quercetagetin binds at the entrance of the RNA template tunnel, a yet unknown binding site for non-nucleoside inhibitors of HCV RdRp. We further showed that quercetagetin acts by inhibiting RNA binding to the RdRp, a new mechanism of action with high potential for pangenotypic activity and a high barrier to resistance. Although quercetagetin is not druggable in its present chemical state, our results open the way to new antiviral approaches for HCV and related viruses that use an RdRp for their replication. Such approaches may use chemical improvement of quercetagetin and/or large-scale structure-based molecule screening and may yield new, highly efficient treatment options for human, animal or plant viral infections.

## SUPPLEMENTARY DATA

Supplementary Data are available at NAR Online.

SUPPLEMENTARY DATA
