# Supplementary material for: Inhibition of RNA binding to hepatitis C virus RNA-dependent RNA polymerase: a new mechanism for antiviral intervention
Source: Nucleic Acids Res. 2014 Jul 22;42(14):9399–409. doi: 10.1093/nar/gku632 (PMC4132742; doi:10.1093/nar/gku632)
Supplement: SUPPLEMENTARY DATA [file supp_42_14_9399__index.html]

Inhibition of RNA binding to hepatitis C virus RNA-dependent RNA polymerase: a new mechanism for antiviral intervention — Inhibition of RNA binding to hepatitis C virus RNA-dependent RNA polymerase: a new mechanism for antiviral intervention — SUPPLEMENTARY DATA 

# Inhibition of RNA binding to hepatitis C virus RNA-dependent RNA polymerase: a new mechanism for antiviral intervention

## SUPPLEMENTARY DATA

**Files in this Data Supplement:**

- SUPPLEMENTARY DATA
- SUPPLEMENTARY DATA
- SUPPLEMENTARY DATA
