# Supplementary material for: Inhibition of RNA binding to hepatitis C virus RNA-dependent RNA polymerase: a new mechanism for antiviral intervention
Source: Nucleic Acids Res. 2014 Jul 22;42(14):9399–409. doi: 10.1093/nar/gku632 (PMC4132742; doi:10.1093/nar/gku632)
Supplement: SUPPLEMENTARY DATA [file supp_gku632_nar-03697-y-2013-File012.pdf]

wwPDB Validation Report

PDB ID: 4OOW
RCSB ID: RCSB084765
TITLE: HCV NS5B polymerase with a fragment of quercetagetin
AUTHOR(S): J.F.Guichou, A.Ahmed-Belkacem, B.Rozenn, N.Nazim, E.Hernandez, C.Pallier, J.M.Pawlotsky

CONFIDENTIAL VALIDATION REPORT

Summary of PDB Entry Validation

The results of the validation of this PDB entry are shown below. No major issues were raised during data processing.

Geometry Validation

1. **Atomic Clashes**

No issues found.

2. **Peptide Linkage**

No issues found.

3. **Covalent Geometry**

No issues found.

4. **Chirality Error**

No issues found.

5. **Phi/Psi Torsion Angles**

No issues found.

CONFIDENTIAL VALIDATION REPORT

Individual Residue Outliers on Real Space R-value

No issues found.

Sequence Validation

The reported biological sequence shows no discrepancy with UniProt sequence (code O92972).

The reported biological sequence and the sequence given in the coordinates show no discrepancy.

Biological Assembly

The biological assembly predicted by PISA is a monomer. This agrees with author's annotation.

Ligand Chemistry

Ligand chemistry has been checked against the Chemical Component Dictionary. The following is a summary.

Identifier: CAQ
Name: CATECHOL
Synonym: 1,2-DIHYDROXYBENZENE
Formula: C6 H6 O2

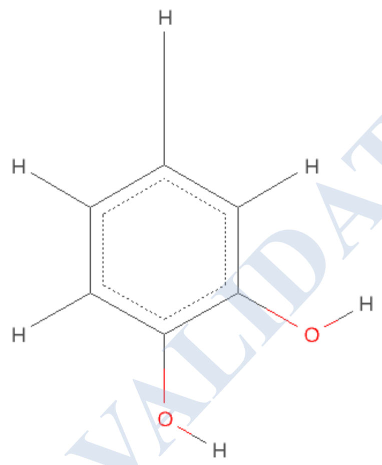

Type	Program	Version	Descriptor
SMILES	ACDLabs	10.04	Oc1ccccc1O
SMILES CANONICAL	CACTVS	3.341	Oc1ccccc1O
SMILES	CACTVS	3.341	Oc1ccccc1O
SMILES CANONICAL	OpenEye OEToolkits	1.5.0	c1ccc(c(c1)O)O
SMILES	OpenEye OEToolkits	1.5.0	c1ccc(c(c1)O)O
InChI	InChI	1.03	InChI=1S/C6H6O2/c7-5-3-1-2-4-6(5)8/h1-4,7-8H
InChIKey	InChI	1.03	YCIMNLLNPGFGHC-UHFFFAOYSA-N

CONFIDENTIAL VALIDATION REPORT

CONFIDENTIAL VALIDATION REPORT

Summary of Structure Factor Validation

Structure quality	
Average Occupancy-weighted avg temperature factor	28.051

Resolution	
High Resolution (Author reported)	2.57
High Resolution (Calculated by REFMAC, V5.7.0029)	2.570
Low Resolution (Author reported)	48.90
Low Resolution (Calculated by REFMAC, V5.7.0029)	84.740

Crystal data	
Space group	P 21 21 21
Total number of reflections	45600
Number of reflections used	43167
Completeness of data	94.7

R-factors	
R-factor (Author reported)	0.216
R-factor (Calculated by REFMAC, V5.7.0029)	0.2152
Free R-factor (Author reported)	0.277
Free R-factor (Calculated by REFMAC, V5.7.0029)	0.2743

Wilson statistics (PHENIX, V1.8-1069)	
Wilson B-factor	32.69
Wilson Scale	0.06

Padilla-Yeates statistics for twin detection (PHENIX, V1.8-1069)	
Padilla-Yeates <|L|>	0.488
Padilla-Yeates <L*L>	0.322
