# Supplementary material for: Inhibition of RNA binding to hepatitis C virus RNA-dependent RNA polymerase: a new mechanism for antiviral intervention
Source: Nucleic Acids Res. 2014 Jul 22;42(14):9399–409. doi: 10.1093/nar/gku632 (PMC4132742; doi:10.1093/nar/gku632)
Supplement: SUPPLEMENTARY DATA [file supp_gku632_nar-03697-y-2013-File010.doc]

**Supplementary Table 1.** Data collection and refinement statistics (molecular replacement)

|  | **Quercetagetin-HCV NS5B**  **(PDB 4OOW)** |
| --- | --- |
| **Data collection** |  |
| Space group | P2_1_2_1_2_1_ |
| Cell dimensions   |  |
| *a*, *b*, *c* (Å) | 106.6, 108.9, 134.8 |
|     α, β, γ  (°) | 90.0, 90.0, 90.0 |
| Resolution (Å) | 48.90 (2.57)* |
| *R*_sym_ or *R*_merge_ | 0.083 (0.336) |
| *I* / σ*I* | 8.2 (2.3) |
| Completeness (%) | 94.7 (90.7) |
| Redundancy | 7.6 (5.6) |
|  |  |
| **Refinement** |  |
| Resolution (Å) | 2.57 |
| No. reflections | 43167 |
| *R*_work_ / *R*_free_ | 21.6/27.7 |
| No. atoms |  |
| Protein | 8611 |
| Ligand | 8 |
| Water | 226 |
| *B*-factors |  |
| Protein | 20.0 |
| Ligand | 53.1 |
| Water | 18.6 |
| R.m.s. deviations |  |
| Bond lengths (Å) | 0.009 |
| Bond angles (°) | 1.29 |

*Values in parentheses are for highest-resolution shell.
