# Supplementary material for: Inhibition of RNA binding to hepatitis C virus RNA-dependent RNA polymerase: a new mechanism for antiviral intervention
Source: Nucleic Acids Res. 2014 Jul 22;42(14):9399–409. doi: 10.1093/nar/gku632 (PMC4132742; doi:10.1093/nar/gku632)
Supplement: SUPPLEMENTARY DATA [file supp_gku632_nar-03697-y-2013-File011.pptx]

## Slide 1
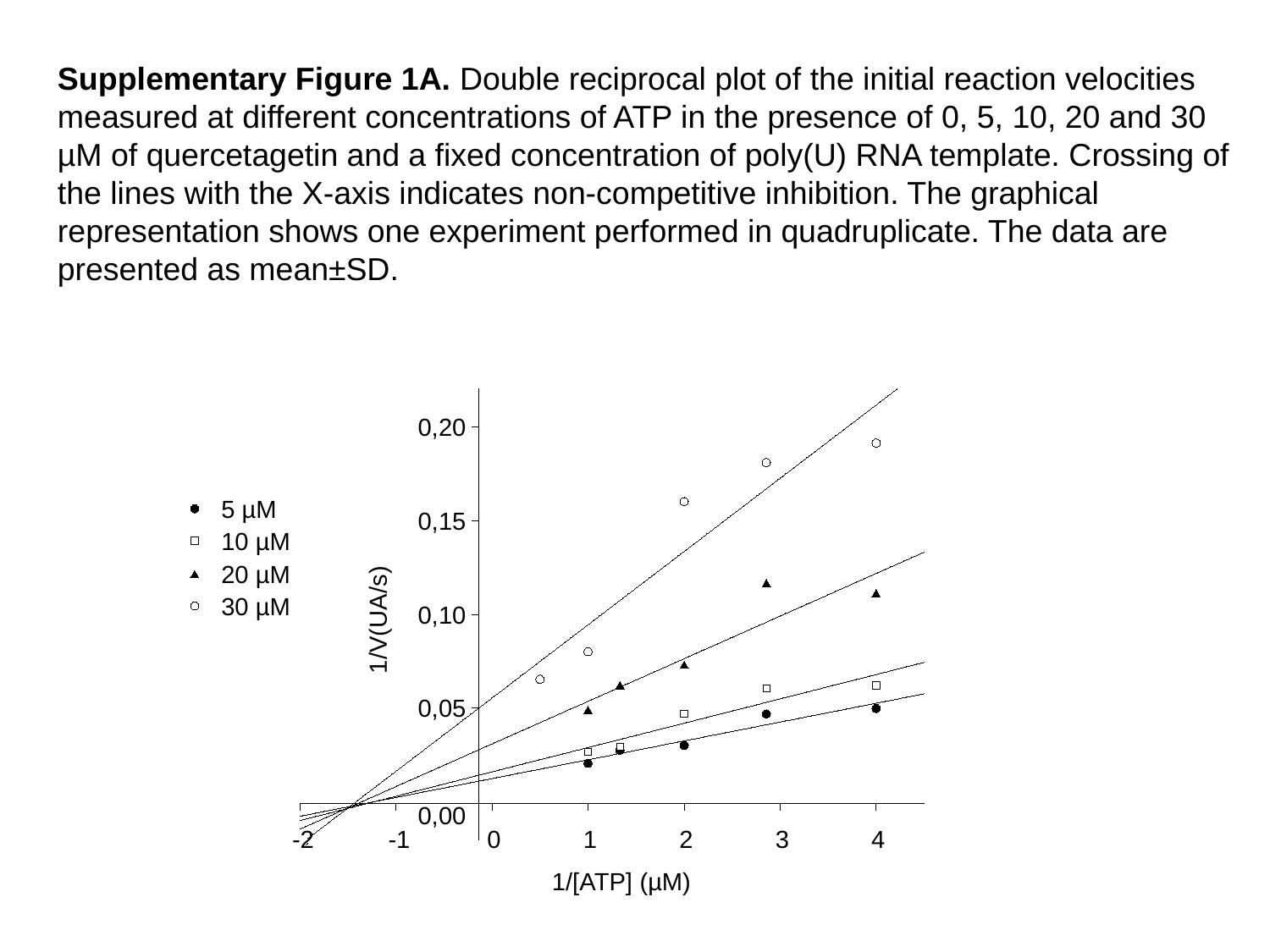

Supplementary Figure 1A. Double reciprocal plot of the initial reaction velocities measured at different concentrations of ATP in the presence of 0, 5, 10, 20 and 30 µM of quercetagetin and a fixed concentration of poly(U) RNA template. Crossing of the lines with the X-axis indicates non-competitive inhibition. The graphical representation shows one experiment performed in quadruplicate. The data are presented as mean±SD.
0,20
5 µM
0,15
10 µM
20 µM
30 µM
0,10
1/V(UA/s)
0,05
0,00
-2
-1
0
1
2
3
4
1/[ATP] (µM)

## Slide 2
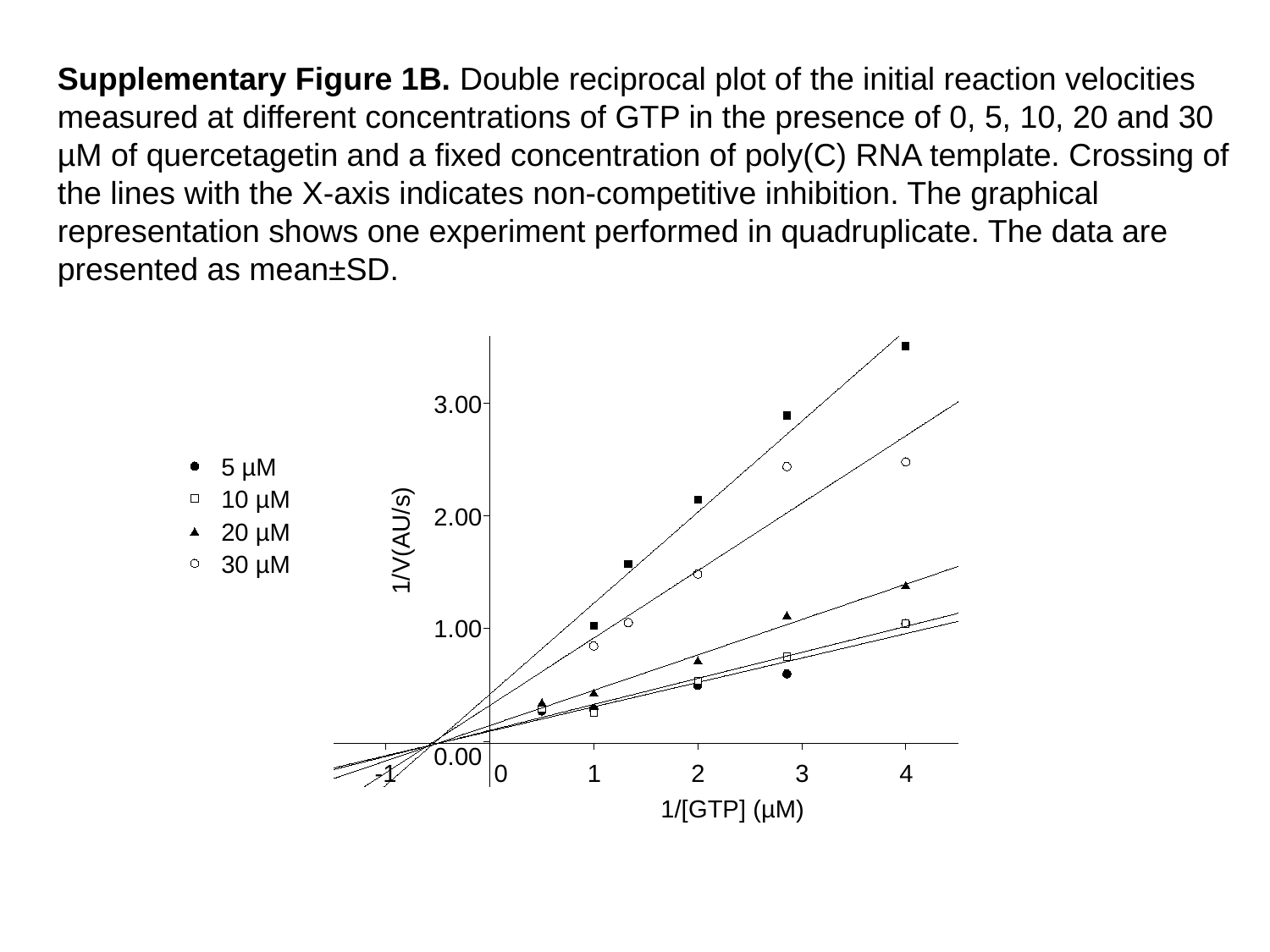

Supplementary Figure 1B. Double reciprocal plot of the initial reaction velocities measured at different concentrations of GTP in the presence of 0, 5, 10, 20 and 30 µM of quercetagetin and a fixed concentration of poly(C) RNA template. Crossing of the lines with the X-axis indicates non-competitive inhibition. The graphical representation shows one experiment performed in quadruplicate. The data are presented as mean±SD.
3.00
5 µM
10 µM
2.00
20 µM
1/V(AU/s)
30 µM
1.00
0.00
-1
0
1
2
3
4
1/[GTP] (µM)

## Slide 3
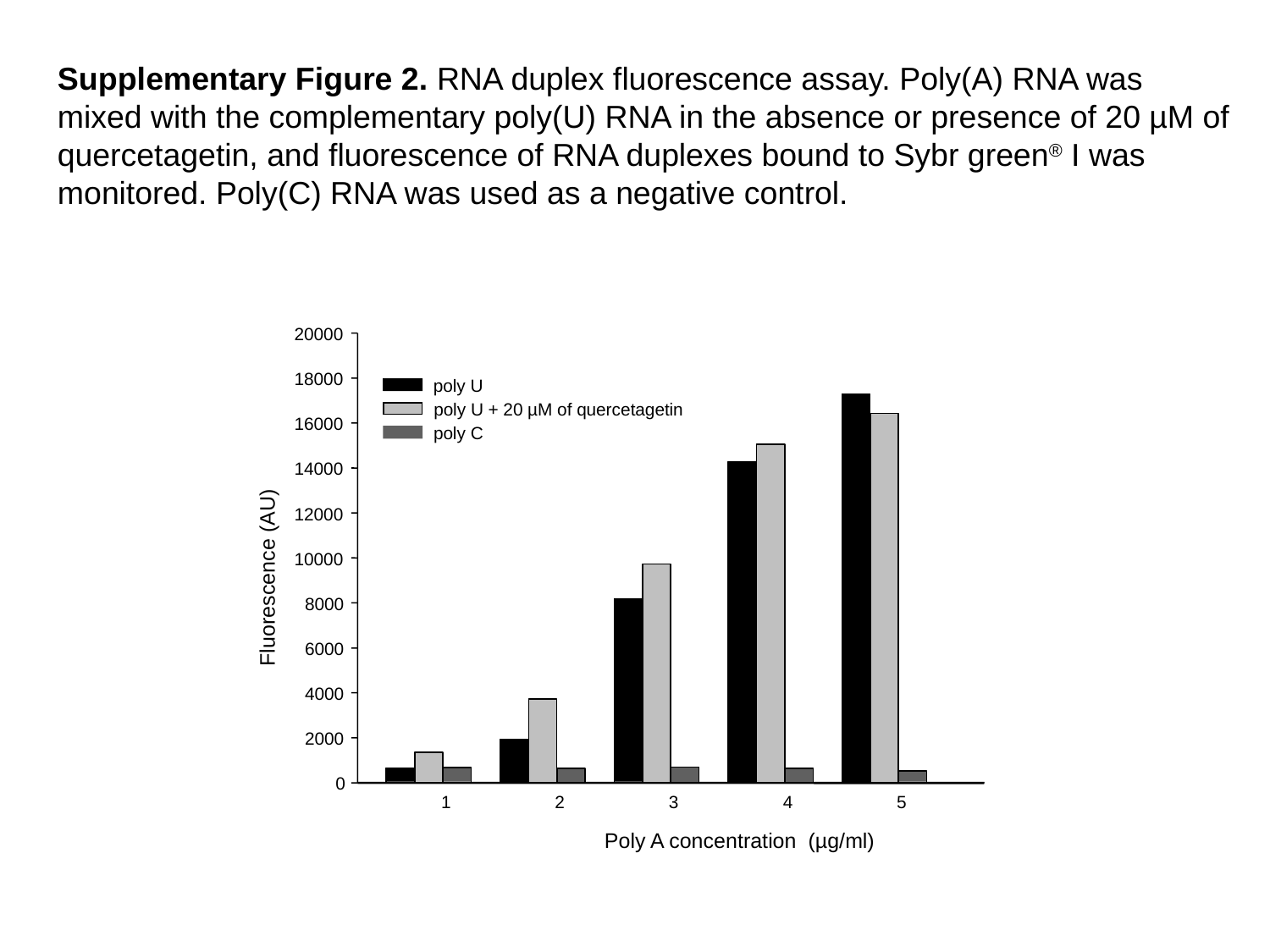

Supplementary Figure 2. RNA duplex fluorescence assay. Poly(A) RNA was mixed with the complementary poly(U) RNA in the absence or presence of 20 µM of quercetagetin, and fluorescence of RNA duplexes bound to Sybr green® I was monitored. Poly(C) RNA was used as a negative control.
20000
18000
poly U
poly U + 20 µM of quercetagetin
16000
poly C
14000
12000
10000
Fluorescence (AU)
8000
6000
4000
2000
0
1
2
3
4
5
Poly A concentration (µg/ml)
